# Immunization of Chickens with Newcastle Disease Virus Expressing H5 Hemagglutinin Protects against Highly Pathogenic H5N1 Avian Influenza Viruses

**DOI:** 10.1371/journal.pone.0006509

**Published:** 2009-08-05

**Authors:** Baibaswata Nayak, Subrat N. Rout, Sachin Kumar, Mohammed S. Khalil, Moustafa M. Fouda, Luay E. Ahmed, Kenneth C. Earhart, Daniel R. Perez, Peter L. Collins, Siba K. Samal

**Affiliations:** 1 Virginia-Maryland Regional College of Veterinary Medicine, University of Maryland, College Park, Maryland, United States of America; 2 Laboratory of Infectious Diseases, National Institute of Allergy and Infectious Diseases, National Institutes of Health, Bethesda, United States of America; 3 Ministry of Environment, Cairo, Egypt; 4 U.S. Naval Medical Research Unit.3, Cairo, Egypt; Institut Pasteur, France

## Abstract

**Background:**

Highly-pathogenic avian influenza virus (HPAIV) and Newcastle disease virus (NDV) are the two most important poultry viruses in the world. Natural low-virulence NDV strains have been used as vaccines over the past 70 years with proven track records. We have previously developed a reverse genetics system to produce low-virulent NDV vaccine strain LaSota from cloned cDNA. This system allows us to use NDV as a vaccine vector for other avian pathogens.

**Methodology/Principal Finding:**

Here, we constructed two recombinant NDVs (rNDVs) each of which expresses the hemagglutinin (HA) gene of HPAIV H5N1strain A/Vietnam/1203/2004 from an added gene. In one, rNDV (rNDV-HA), the open reading frame (ORF) of HA gene was expressed without modification. In the second, rNDV (rNDV-HAF), the ORF was modified so that the transmembrane and cytoplasmic domains of the encoded HA gene were replaced with those of the NDV F protein. The insertion of either version of the HA ORF did not increase the virulence of the rNDV vector. The HA protein was found to be incorporated into the envelopes of both rNDV-HA and rNDV-HAF. However, there was an enhanced incorporation of the HA protein in rNDV-HAF. Chickens immunized with a single dose of either rNDV-HA or rNDV-HAF induced a high titer of HPAIV H5-specific antibodies and were completely protected against challenge with NDV as well as lethal challenges of both homologous and heterologous HPAIV H5N1.

**Conclusion and Significance:**

Our results suggest that these chimeric viruses have potential as safe and effective bivalent vaccines against NDV and. HPAIV. These vaccines will be convenient and affordable, which will be highly beneficial to the poultry industry. Furthermore, immunization with these vaccines will permit serological differentiation of vaccinated and avian influenza field virus infected animals.

## Introduction

Avian Influenza (AI) is an economically-important disease of poultry worldwide. The etiologic agent is a segmented negative-strand RNA virus in the genus Influenza virus A of the family *Orthomyxoviridae*. Avian influenza virus (AIV) strains are divided into subtypes based on serological reactions of the two surface glycoproteins, the hemagglutinin (HA) and neuraminidase (NA). There are 16 different HA (H1-H16) and 9 different NA (N1-N9) subtypes [1], [2]. In addition, AIV strains are further classified into two different pathotypes, low pathogenic (LP) and highly pathogenic (HP). Historically, HPAIV of subtypes H5 and H7 have caused severe disease and high mortality in poultry. Since 1996, HPAIV strains of subtype H5N1 have emerged recently in Asia have caused disease in poultry around the world, resulting in the culling or death of more than 500 million poultry in more than 62 countries. H5N1 HPAIV also has caused zoonotic infections in humans, resulting in 406 confirmed cases and 254 deaths to date [3], [4]. The transmission of H5N1 HPAIV from birds to humans remains a serious threat to public health. Therefore, control of H5N1 virus infections in poultry is not only important for the poultry industry, but also for preventing H5N1 infection in humans.

Traditionally, HPAI is controlled by elimination of infected flocks. Due to economical reasons, culling of infected flocks is no longer a practical method for control of AI in either developed or developing countries. Although vaccination has been recommended by the World Organization for Animal Health (OIE) and the Food and Agriculture Organization (FAO) to control AI, few effective AI vaccines are available [5]. For the most part, these consist of inactivated preparations of whole influenza viruses. However, inactivated vaccines are not commonly used because of the high cost and the difficulty in “differentiating infected from vaccinated animals” (DIVA). Live attenuated vaccines are not used because of concern that the vaccine viruses may, through either mutation or genetic reassortment with circulating strains, become virulent [6]. To overcome these difficulties, several strategies have been developed based on *in vivo* expression of HA by DNA vaccines [7], [8] or by vectors based on adenovirus [9], [10], [11], fowl pox virus [12], [13], [14], [15], baculovirus [16], [17], [18], infectious laryngotracheitis virus (ILTV) [19], [20], or Newcastle disease virus (NDV) [21], [22], [23], [24].

Of all AI vaccine strategies, HA protein expressed *in vivo* using NDV as a vector has shown the most promising results [21], [22], [23]. NDV is a non-segmented, negative-strand RNA virus belonging to genus *Avulavirus* in the family *Paramyxoviridae*. Virulent NDV strains cause a severe respiratory and neurologic disease in poultry world wide. Naturally-occurring avirulent NDV strains have been successfully used as vaccines for more than 70 years. Development of reverse genetic systems has allowed manipulation of the genome of NDV [25], [26], [27] and generation of recombinant NDVs that express foreign proteins from added genes [24], [28], [29]. Several groups have used NDV reverse genetic systems to develop vaccines against AIV subtypes H5 [21], [23] and H7 [22].

There have been two independent studies to develop bivalent vaccines against NDV and HPAI H5 virus [21], [23], with different levels of success. In one study, the HA gene from an H5N2 virus was inserted between the F and HN genes of NDV [23]. This recombinant NDV protected chickens against both NDV and H5N2 virus, but some vaccinated chickens showed signs of clinical disease when challenged with highly- pathogenic H5N2 virus. In the other study, the HA gene from an H5N1 virus was inserted between the P and M genes of NDV [21]. This recombinant NDV protected chickens against both NDV and two different strains of H5N1 viruses isolated in China.

In this study, we have attempted to develop improved bivalent vaccines against NDV and HPAI H5 virus by using recombinant NDV (rNDV) vaccine strain LaSota as a vector to express the HA protein from an HPAI H5N1 virus. Two different rNDVs were constructed expressing different forms of the HA protein: one vector (rNDV-HA) expressed an unmodified form of HA, while the other (rNDV-HAF) expressed a version of HA in which the HA ectodomain was fused with the transmembrane and cytoplasmic domains derived from the F protein of NDV. This resulted in a chimeric HA protein with enhanced incorporation of foreign proteins into NDV particles. The protective efficacies of both these bivalent vaccines were compared in chickens by challenge with a lethal dose of highly-virulent NDV and homologous and heterologous H5N1 HPAIV. Our results showed that a single immunization with either of the candidate bivalent vaccines completely protected against both pathogens in chickens. A further enhanced incorporation of the H5 HA into NDV particles was observed in rNDV-HAF vaccine than into rNDV-HA vaccine, indicating a better ability of this virus to induce neutralization antibody response in chickens.

## Results

### Generation of rNDVs expressing two versions of the HA protein of H5N1 AIV

**Figure 1:**
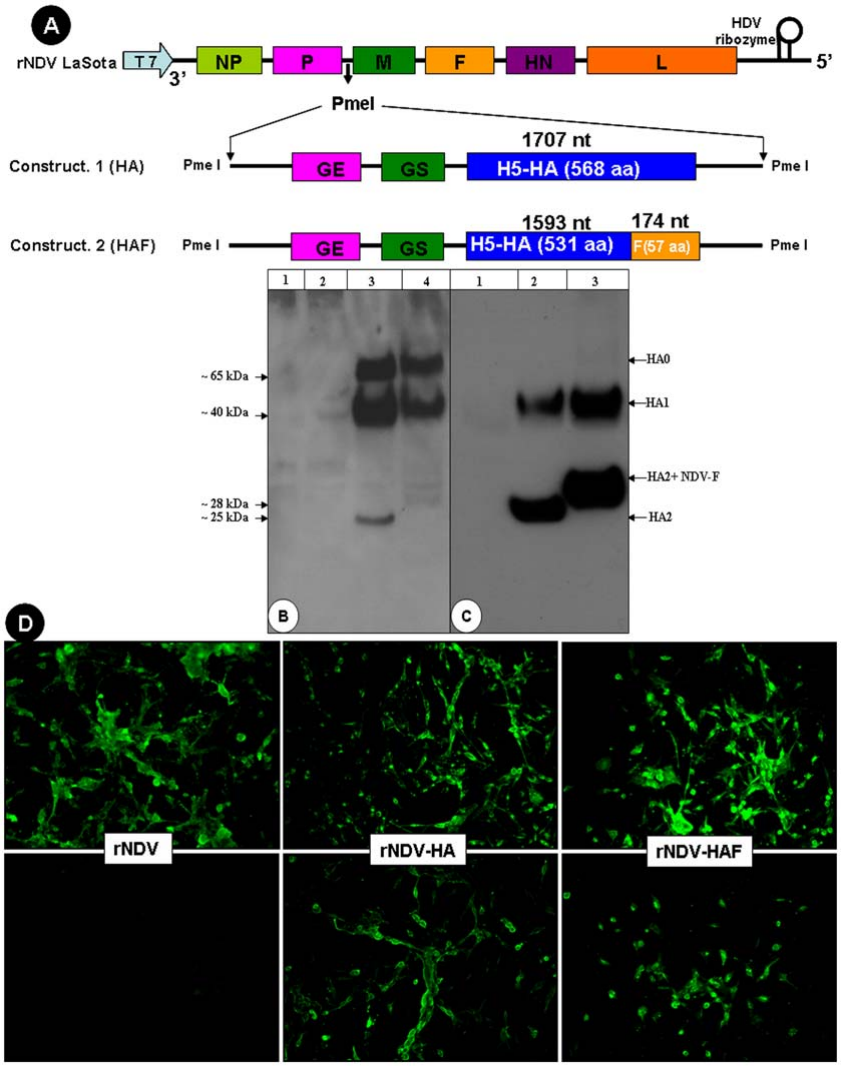
Construction and characterization of recombinant NDVs expressing unmodified or chimeric versions of the H5 HPAIV HA protein. (A) Schematic diagram depicting the rNDV LaSota genome with insertion of an added gene engineered to express unmodified HA (construct 1) or chimeric HA consisting of the HA ectodomain fused to the transmembrane and cytoplasmic domains of the NDV F protein (construct 2). In the inserted sequence, the foreign HA coding sequence was preceded by an NDV gene junction containing gene-end (GE) and gene-start (GS) transcription signal: this results in the HA ORF being under the control of a set of transcription signals such that it is expressed as a separate mRNA. (B) Expression and processing of H5 HA and chimeric HA in DF1 cells infected with the respective rNDVs and analyzed by by Western blot analysis using chicken polyclonal H5N1 AIV antibodies. HA specific bands were not detected in lysates of mock-infected DF1 cells (Lane 1) or cells infected with rNDV empty vector (Lane 2). In contrast, lysates of cells infected with rNDV-HA (Lane 3) or rNDV-HAF (Lane 4) contained uncleaved inactive precursor (HA0) and proteolytically cleaved HA1 and HA2/HA2-F proteins. (C) Incorporation of the H5 HA protein in purified rNDV particles, detected by Western blot analysis. HA-specific bands corresponding to the cleaved HA1 and HA2/HA-F2 species were detected in preparations of purified particles of rNDV-HA (Lane 2) and rNDV-HAF (Lane 3), but not the empty vector rNDV (lane 1). (D) Immunofluorescence analysis of DF 1 cells infected with rNDV, rNDV-HA and rNDV-HAF and processed for intracellular expression of NDV proteins (upper panels) and H5 HA protein (lower panel), detected using chicken polyclonal anti-NDV and anti-H5N1 AIV antibodies.

Two different rNDVs were constructed expressing different versions of the HA protein of HPAIV strain A/Vietnam/1203/2004, a virus isolated from a fatal human infection [30]. In each construct, the HPAIV coding sequence was placed under the control of NDV transcriptional signals and inserted, by reverse genetics, between the P and M genes in the NDV LaSota avirulent vaccine strain (Figure 1A). One construct, rNDV-HA, was designed to express the unmodified HA protein; the other (rNDV-HAF), was designed to express a chimeric HA protein in which the ectodomain of HA was fused with the transmembrane domain and cytoplasmic tail region of the NDV F protein (Figure 1A, constructs 1 and 2, respectively). Both recombinant viruses were recovered using the reverse genetics method described previously [31]. The structure of each HA insert in the genome of these viruses was confirmed by RT-PCR and nucleotide sequence analysis (data not shown).

### Expression of the native and chimeric forms of the HA protein by rNDV

The expression of the two versions of the H5 HA protein in DF1 cells infected with rNDV-HA and rNDV-HAF were analyzed by Western blot using polyclonal chicken H5N1 AIV antiserum (Figure 1B). Immunoblot analysis detected three bands in lysates of cells infected with rNDV-HA or rNDV-HAF: these represented (i) the inactive HA precursor HA0, with an apparent molecular weight of ∼65,000 for rNDV-HA (Figure 1B, Lane 3), and ∼66,000 for rNDV-HAF (Figure 1B, Lane 4), (ii) the N-terminally-derived proteolytic cleavage product HA1 with an apparent molecular weight of ∼40,000 for both viruses, and (iii) the C-terminally-derived proteolytic cleavage HA2 of apparent molecular weight ∼25,000 for rNDV-HA and ∼28,000 for rNDV-HAF. The differences in apparent size reflected the different transmembrane and cytoplasmic domains. As expected, HA was not detected in DF1 cells that were mock-infected (Figure 1B, Lane 1) or infected with rNDV empty vector (Figure 1B, Lane 2).

We also used immunofluorescence to localize expression of the native and chimeric HA proteins in DF-1 cells infected with the recombinant viruses. Intracellular expression was investigated in cells that were fixed and permeabilized with Triton detergent (Figure 1D). Positive staining was observed for cells infected with rNDV-HA or rNDV-HAF using chicken antiserum for NDV (Figure 1D, upper panel) or H5N1 AIV (Figure 1D, lower panels).

### Incorporation of AIV HA protein into the rNDV virion

**Figure 2:**
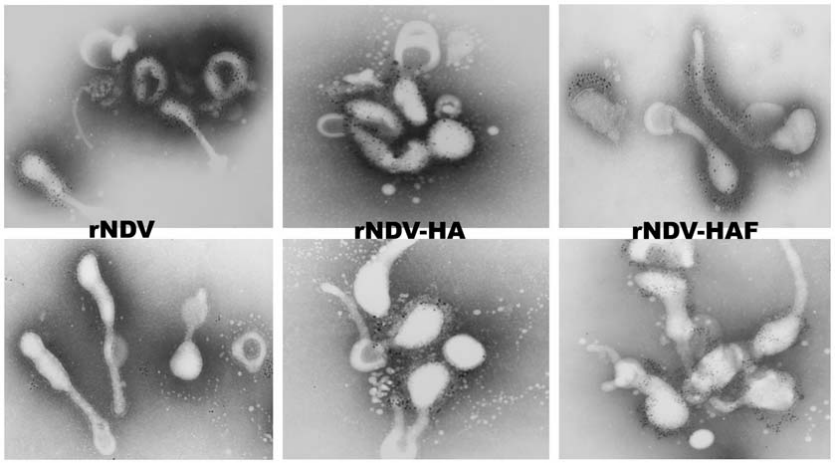
Immunoelectron microscopy of purified virions of rNDV, rNDV-HA and rNDV-HAF, analyzed using mouse monoclonal antibodies against the NDV HN protein (upper panel) or chicken polyclonal antibodies against H5N1 AIV (lower panel).

The recombinant viruses (rNDV, rNDV-HA and rNDV-HAF) were partially purified from allantoic fluid by sucrose density gradient centrifugation. Incorporation of AIV HA into the rNDV envelope was analyzed by Western blot assay (Figure 1C) and immunogold electron microscopy (Figure 2). Western blot analysis of purified viruses with anti H5N1 AIV antibodies did not detect any bands for rNDV, as expected (Figure 1C, Lane 1), whereas HA1 and HA2 bands were detected in both rNDV-HA and rNDV-HAF (Figure 1C, Lanes 3 and 2). The inactive precursor HA0 that was detected in infected cells (Figure 1B) was not detected in purified viruses (Figure 1C). There were two-fold more HA1 and HA2 proteins in virions of rNDV-HAF, compared to rNDV-HA, as determined by densitometry analysis of the bands. The band intensities were normalized against NDV HN protein as an endogenous control. This suggested that fusion of the transmembrane domain and cytoplasmic tail region of NDV F protein with the HA ectodomain enhanced incorporation of HA protein into NDV particles. This was consistent with the results of immunoelectron microscopy (Figure 2). Antiserum to NDV directed the deposition of gold particles on the envelopes of rNDV, rNDV-HA, and rNDV-HAF, as expected (Figure 2, upper panels). Antiserum to H5N1 AIV directed deposition to the envelopes of rNDV-HA and rNDV-HAF (Figure 2, lower panel). As expected, there was no gold particle deposit on the envelope of rNDV. There was significantly more gold particle deposit on the envelope of rNDV-HAF than on rNDV-HA with same amount of anti H5N1 AIV antibodies (Figure 2, lower panel). This suggested that the chimeric version of the HA protein was incorporated more efficiently into the NDV envelope than the native HA protein.

### Biological characterization of rNDV expressing the native or chimeric HA protein

**Figure 3:**
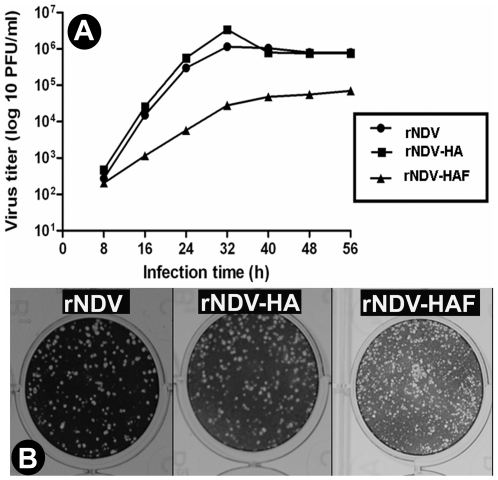
Multicycle growth kinetics (A) and plaque morphology (B) of the rNDV, rNDV-HA and rNDV-HAF viruses in DF-1 cells.

The multicycle growth kinetics of rNDV-HA and rNDV-HAF were compared with those of the parental rNDV. Our results demonstrated similar kinetics of growth between rNDV and rNDV-HA, whereas rNDV-HAF grew more slowly and achieved a maximal titer that was approximately one log lower than those of parental rNDV titers (Figure 3A). Similar plaque sizes were observed for rNDV and rNDV-HA, but a slightly smaller plaque size was observed for rNDV-HAF (Figure 3B). The pathogenicities of wild type NDV strain LaSota (wt LaSota) and parental rNDV, rNDV-HA and rNDV-HAF were evaluated by the mean death time (MDT) test in embryonated chicken eggs. The MDTs for these viruses were 105 h (wt NDV), 110 h (rNDV), 112 h (rNDV-HA) and>120 h (rNDV-HAF). According to OIE guidelines, an NDV strain is considered lentogenic or avirulent, if the MDT value is>90 h [32], and thus the two rNDVs expressing the two versions of HA are avirulent viruses. Indeed, the presence of the added native or chimeric HA gene conferred attenuation to the NDV vector.

### Immunogenicity and protective efficacy in chickens against highly-virulent NDV challenge

To evaluate protective efficacy, groups of chickens were inoculated by the oculonasal route with wt NDV LaSota (the naturally attenuated vaccine strain that was the parent for the NDV vectors), or with the recombinant rNDV-HA and rNDV-HAF viruses, or were left unimmunized as a control group (Table 1). The birds were challenged 21 days later by the oculonasal route with highly-virulent NDV strain Texas-GB. All of the chickens that had been immunized with biologically-derived wt LaSota, rNDV-HA, or rNDV-HAF were completely protected from NDV challenge, with no evidence of virus shedding and without any disease signs (Table 1). In contrast, all of the chickens in the unvaccinated control group died within 3 days after challenge (Table 1). Each of the NDV immunizations induced a substantial titer of NDV-specific HI serum antibodies when assayed on day 21 prior to challenge. However, the mean antibody titers induced by rNDV-HA and rNDV-HAF were approximately one and two log_2_ less than that of wt NDV, respectively (Table 1). This modest reduction in immune response to the vector might reflect increased attenuation of the rNDVs associated with the insertion of the HA gene, as indicated above by the MDT assay, or might reflect an effect on expression of the NDV HN protein (the antigen-specificity of the antibodies measured by the HI assay) due to the presence of the upstream foreign gene, or both.

**Table 1:** NDV-specific serum antibody responses following immunization with NDV-HA or NDV-HAF and survival of chickens following virulent NDV strain Texas-GB challenge^1^.

	Response to immunization		Protection of chickens post challenge^2^		
Chicken group	No. of birds seropositive	Mean NDV serum antibody HI titer (log_2_)	Oral shedding	Cloacal shedding	No. of survivors
**Uninfected control**	0/10	0.00	10/10	4/10	0/10
**wtNDV (LaSota)**	10/10	6.18±0.91	0/10	0/10	10/10
**rNDV-HA**	10/10	5.50±0.70	0/10	0/10	10/10
**rNDV-HAF**	10/10	4.10±0.63	0/10	0/10	10/10

1 Chickens in groups of 10 were vaccinated on day 0 with wtNDV, rNDV-HA, or rNDV-HAF. On day 21, serum samples were taken and the birds were challenged with 10^4^ ELD_50_ of virulent NDV strain Texas GB.

2 Shedding of challenge virus was determined by inoculation of oropharyngeal (oral) and cloacal swab samples into of embryonated chicken eggs followed by HA tests of allantoic fluid. All of the uninfected control birds died by day 3, and oral and cloacal samples were taken at the time of death. For the other birds, samples were taken on day 3.

### Protective efficacy in chickens against homologous and heterologous HPAIV challenge

As a result of genetic drift, there exists considerable antigenic diversity in the HA protein among H5N1 viruses, resulting in distinct clades and subclades within the HA of the H5N1 lineage viruses. Currently, two clades are circulating among poultry, namely clade 1 that includes the A/Vietnam/1203/2004 strain represented in the NDV vectors, and clade 2.2 that includes the A/Egret/Egypt/01/2006 virus. Therefore, we were interested in evaluating the ability of the NDV-HA and -HAF vectors to provide protection against challenge with the clade 1 and clade 2.2 HPAIV. Groups of chickens were vaccinated with rNDV, rNDV-HA, rNDV-HAF, or were left unvaccinated. The pre-challenge sera collected at 21 days post-vaccination had mean NDV HI antibody titers (log_2_) of 5.30±0.94, 5.15±0.95 and 4.50±0.70 for rNDV, rNDV-HA and rNDV-HAF, respectively (Table 2). Thus, the reduction in NDV-specific antibodies associated with the presence of the foreign HA gene was less than in the preceding experiment shown in Table 1. The mean HPAIV HI serum antibody titers (log_2_) in birds immunized rNDV, rNDV-HA or rNDV-HAF were 0, 4.57±1.06 and 4.53±1.36, respectively, against the homologous A/Vietnam/1203/2004 virus, and were 0, 2.92±1.09, and 2.38±1.35, respectively, against the heterologous A/Egret/Egypt/01/2006 virus (Table 2). The rNDV-HA- and rNDV-HAF-vaccinated birds were completely protected from intranasal challenge by 100 CLD_50_ doses of either the homologous or heterologous HPAIV, with no clinical disease signs or deaths (Table 2). In contrast, all of the chickens in the unvaccinated control and rNDV-vaccinated (empty vector) groups died within 24–48 h following challenge with the A/Vietnam/1203/2004 virus, and within 48–72 h following challenge with the A/Egret/Egypt/01/2006 virus. The birds that died from the latter virus had signs of greenish white diarrhea and hemorrhagic signs in the legs and combs.

**Table 2:** NDV- and HPAIV-specific serum antibody responses following immunization with rNDV-HA and rNDV-HAF and survival of chickens after homologous and heterologous HPAIV challenge.

	Response to immunization (samples taken on 21 day)			
Groups: Immunizing virus/Challenge HPAIV (no. of birds)^1^	Mean NDV serum HI antibody (log_2_)	No. of birds seropositive to the indicated HPAIV	Mean serum HI antibody (log_2_) to indicated HPAIV	No. of survivors following challenge on day 21 with the indicated HPAIV
**Uninfected/Viet (5)**	(0)^2^	0/5	0	0/5
**Uninfected/Egypt (5)**		0/5	0	0/5
**rNDV/Viet (5)**	(5.30±0.94)^2^	0/5	0	0/5
**rNDV/Egypt (5)**		0/5	0	0/5
**rNDV/Viet (10)**	(5.15±0.95)^2^	10/10	4.57±1.06	10/10
**rNDV/Egypt (10)**		10/10	2.92±1.09	10/10
**rNDV/Viet (10)**	(4.50±0.70)^2^	10/10	4.53±1.36	10/10
**rNDV/Egypt (10)**		10/10	2.38±1.35	10/10

1 Chickens in groups of 5 or 10, as indicated, were vaccinated on day 0 with wtNDV, rNDV-HA, or rNDV-HAF. On day 21, serum samples were taken and the birds were challenged with 100 CLD_50_ of the indicated HPAIV. Groups are named according to the immunizing NDV vector and the HPAIV strain used in the HI assays and challenge. Viet = homologous A/Vietnam/1203/2004; Egypt = heterologous A/Egret/Egypt/01/2006.

2 Mean of the viruses in #1 and #2.

### Shedding of vaccine and challenge viruses in infected chickens

Shedding of NDV vaccine and HPAIV challenge viruses was monitored by taking oral and cloacal swab samples on day 3 post-immunization and days 3, 5, and 7 post-challenge. The presence of infectious NDV vector or HPAIV challenge virus was determined by inoculation of embryonated chicken eggs and confirmation of virus replication by HA assay. No cloacal shedding was observed for any of the NDV vaccine viruses post-immunization, consistent with the attenuated nature of the NDV vector (Table 3). All of birds immunized with rNDV and rNDV-HA were positive for oral shedding, but only 14 out of 20 birds immunized with rNDV-HAF were positive for oral shedding. Analysis of shedding of NDV vaccine virus was performed to confirm immunization and its replication in chickens. Although the exact time at which vaccine virus stopped shedding was not determined, NDV lentogenic vaccine strains known to shed 5 days post immunization. Following challenge with homologous or heterologous HPAIV, no HPAIV shedding was observed in rNDV-HA- and rNDV-HAF-immunized birds at 3, 5 and 7 days post-challenge. In contrast, both oral and cloacal shedding of challenge viruses were observed in all (oral) or most (cloacal) of the unvaccinated and rNDV (empty vector) -vaccinated birds, respectively, of the challenge virus. The shedding results of only day 3 post-challenge are shown in Table 3.

**Table 3 pone-0006509-t003:** Frequency of shedding of the NDV-based vaccine viruses and HPAIV challenge viruses^1^.

	rNDV-based vaccine virus		HPAIV challenge virus			
			A/Vietnam/1203/2004		A/Egret/Egypt/01/2006	
Chicken groups	Oral	Cloacal	Oral	Cloacal	Oral	Cloacal
**Uninfected control**	-	-	5/5	4/5	5/5	4/5
**rNDV**	10/10	0/10	5/5	4/5	5/5	3/5
**rNDV HA**	20/20	0/10	0/10	0/10	0/10	0/10
**rNDV HAF**	14/20	0/10	0/10	0/10	0/10	0/10

1 From the experiment in Table 2. Shedding was determined by collecting oral and cloacal swabs on 3 day post vaccination and 3 day post challenge and inoculation into embryonated chicken eggs followed by HA tests for virus growth.

### Comparison of pre- and post-HPAIV challenge serum antibody responses by the DIVA strategy

The pre-challenge sera collected from birds at 21 days post-immunization with rNDV-HA and rNDV-HAF were compared with sera collected at 10 days post-challenge with HPAI A/Vietnam/1203/2004 or A/Egret/Egypt/01/2006 viruses. Unvaccinated control and rNDV (empty vector) vaccinated chickens did not survive the HPAIV challenge, hence pre- and post challenge comparison was not carried out. The comparison was carried out using a commercially available AIV NP ELISA that provides the basis to differentiate between birds vaccinated with an NDV-based vector (which would not stimulate antibodies against HPAIV NP) and infected birds (in which an NP-specific response would occur). Pre- and post-challenge sera also were analyzed by a commercial NDV ELISA test and an HI assay using the respective homologous or heterologous HPAIV strain. There was little or no change in NDV antibody titers detected by NDV ELISA following HAPIV challenge, as might be expected (Table 4). However, in both rNDV-HA- and rNDV-HAF- vaccinated birds, the HPAIV HI titers were increased by one and two log_2_ units following A/Vietnam/1203/2004 and A/Egret/Egypt/01/2006 challenge, respectively. However, both the pre- and post-challenge sera of rNDV-HA- and rNDV-HAF-vaccinated birds were negative for AIV NP antibody by AIV NP ELISA. The lack of a detectable response to the AIV NP protein following HPAIV challenge suggested that there was little or no challenge virus replication.

**Table 4 pone-0006509-t004:** Comparison of pre-challenge (day 21) and post-challenge (day 31) NDV- and HPAIV-specific serum antibody titers^1^.

Groups: Immunizing virus/Challenge HPAIV (no.of birds)^2^	Mean NDV serum ELISA titer2a		Mean serum HI antibody (log_2_) to the indicated HPAIV^3^		Mean HPAIV NP ELISA titer (OD)^4^	
	Pre-	Post-	Pre-	Post-	Pre-	Post-
**rNDV-HA/Viet (10)**	1.39±0.38	1.39±0.38	4.40±0.96	5.80±0.78	0.074±0.012	0.082±0.013
**rNDV-HA/Egypt (10)**	1.25±0.37	1.22±0.23	2.90±1.10	4.90±1.42	0.071±0.008	0.084±0.007
**rNDV-HAF/Viet (10)**	1.25±0.37	1.19±0.46	4.45±1.78	5.18±1.71	0.071±0.004	0.088±0.033
**rNDV-HAF/Egypt (10)**	1.12±0.42	1.10±0.16	2.80±1.61	4.90±0.73	0.068±0.005	0.095±0.033

1 Continuation of the experiment in Tables 2 and 3.

2 Viet = A/Vietnam/1203/2004; Egypt = A/Egret/Egypt/01/2006.

2a Commercial NDV ELISA assay (Synbiotic Corporation, San Diego, CA).

3 The challenge virus was used for the HI antibody assays.

4 Commercial HPAIV NP ELISA assay (Synbiotic Corporation, San Diego, CA). Negative control OD = 0.083 and Positive control OD = 0.867.

### Neutralization of diverse HPAIV strains with sera from birds immunized with NDV-HA or NDV-HAF

The ability of sera from vaccinated chickens (from the study described in Tables 2–4) to neutralize homologous and heterologous strains of HPAIV was assessed by virus neutralization (VN) and HI tests (Table 5). Both rNDV-HA and rNDV-HAF elicited serum antibodies that reacted with H5N1 clade 1 viruses (A/Vietnam/1203/2004, A/Hong Kong/213/2006), clade 2.2 virus (A/Egret/Egypt/01/2006) and an H5N2 virus isolated from chickens (A/Pennsylvania/8/3/83) by HI tests, and which neutralized each of these viruses by VN tests. This result indicated that the antibodies induced by rNDV-HA and rNDV-HAF have abilities to neutralize a broad range of HPAI H5 subtype viruses.

**Table 5:** Serum antibodies induced by NDV-HA or NDV-HAF: HI and virus-neutralizing (VNT) antibody titers against homologous and heterologus HPAIV strains.

	Serum antibody titers against the indicated HPAIV strains			
Immunizing virus	Vietnam/1203/2004 (H5N1)	Egret/Egypt/2006 (H5N1)	Hongkong/213/2006 (H5N1)	Pennsylvania/8/3/1983 (H5N2)
**Mean HPAIV HI titer (log_2_)** 1				
**rNDV**	0	0	0	0
**rNDV- HA**	4.57±1.06	2.92±1.09	5.19±0.93	2.98±1.28
**rNDV- HAF**	4.53±1.36	2.38±1.42	5.19±1.23	2.96±1.61
**Mean VNT titer (log_2_)** 1				
**rNDV**	0	0	0	0
**rNDV- HA**	5.19±1.23	2.60±0.99	6.78±0.93	5.23±0.81
**rNDV- HAF**	5.30±1.46	3.19±1.47	6.87±1.07	5.42±1.23

1 Sera are from the experiment in Table 2, taken 21 days following a single immunization with the indicated virus expressing the HA of the Vietnam/1203/2004 strain. The numbers of animals in the groups were: rNDV (n = 10), rNDV-HA (n = 20), rNDV-HAF (n = 20). Note that the text uses VN for virus neutralization, rather than VNT.

## Discussion

In the past twelve years, HPAI H5N1 viruses have not only devastated poultry industries but also have become a major public health concern [3], [4].These viruses have shown continuous increases in genetic and antigenic heterogeneity, as well as dissemination in Asia, Africa and Europe. In order to limit the infection of H5N1 viruses in poultry, there is a need to develop vaccines that provide broad and effective immunity and protection against these viruses. One vaccine that has shown promising results is based on using NDV as a vector to express the HPAIV HA protein from an added gene [21], [22], [23]. Newcastle disease is an economically important disease of poultry, and vaccination of poultry with live NDV vaccines is readily achieved and is mandatory in many countries. Therefore, development of an efficacious live bivalent vaccine against AIV and NDV has several advantages, including price per dose, ease of administration, and ability to differentiate infected from vaccinated animals.

Our goal is to develop NDV as a vaccine vector for protection of birds against HPAIV H5 subtype virus infections. Previously, two research groups have published results describing generation of NDV recombinant vaccines against HPAI H5 subtype virus [21], [23]. In the present study, we have generated improved recombinant NDV vaccines that provide greater protection in chickens against homologous and heterologous HPAI H5 virus challenges than those previously observed. Veits *et al.*, [23] generated an rNDV in which the HA gene from an H5N2 virus was inserted between F and HN genes of NDV clone 30, a derivative of strain LaSota. Although their rNDV protected chickens from lethal NDV and highly-pathogenic H5N2 virus challenges, some vaccinated chickens showed signs of disease when challenged with H5N2 virus, indicating only partial protection. Ge *et al.*,[21] constructed an rNDV in which the HA gene from an HPAI H5N1 virus isolated from an wild bird in China was inserted between the P and M genes of NDV strain LaSota. The recombinant virus protected chickens from the disease following challenges with NDV and homologous and heterologous H5N1 HPAIV. However, the heterologous challenge virus was detected from the lung, spleen, kidney and brain of one of three chickens at 3 days post-challenge. In that study, both the challenge viruses used were selected from the same geographic area and having 95% similarity in HA gene. Therefore, there is a need to improve NDV-vectored AIV vaccines to provide complete protection against homologous and heterologous challenge viruses isolated from different geographic areas.

In our study, we have generated two different rNDV expressing the H5 HA protein. In one rNDV, the HA gene was expressed in native, unmodified form, while in the other it was modified to contain the transmembrane and cytoplasmic region of NDV F protein in place of its own. This was done with the idea that this would provide for enhanced incorporation into the NDV particle. The unmodified and modified HA genes were inserted separately between the P and M genes of NDV strain LaSota. Both of the recombinant vaccine viruses induced high levels of serum antibody that efficiently neutralized NDV as well as four different strains of HPAI H5 subtype viruses, which included three H5N1 viruses isolated from a humans and one H5N2 virus isolated from a poultry outbreak in the U.S. Both of the recombinant viruses completely protected chickens against otherwise-lethal NDV and HPAI H5N1 virus challenges. The challenge viruses used were isolated from Vietnam and Egypt. None of the vaccinated animals shed detectable amounts of challenge virus, demonstrating the efficacy of our vaccines. Furthermore, none of the vaccinated chickens had a detectable serum antibody response to the NP protein of AIV after 10 days of challenge, suggesting that there was little or no challenge virus replication in vaccinated chickens.

An ideal AI vaccine should elicit robust protective immune responses against a broad range of homologous and heterologous H5 influenza strains. Therefore, it is necessary to select the HA gene from a virus that can represent most H5 strains of that nature. In our study, we chose the HA gene from A/Vietnam/1203/2004 virus for generation of recombinant NDV. This H5N1 virus belongs genetically to clade 1 and persists in Asia. The heterologous H5N1 challenge virus A/Egret/Egypt/01/2006 used in this study belongs to clade 2.2 and persists in Africa. There is approximately 7% amino acid divergence between the HA1 portions of the two viruses [33]. Single vaccinations of rNDV-HA or -HAF completely protected chickens against both clade 1 and clade 2.2 challenge viruses. Analysis of the pre-challenge serum neutralizing antibody responses of vaccinated chickens to H5N1 viruses showed that there was higher neutralizing activity against the homologous clade 1 virus compared to the heterologous clade 2.2 virus, which was an indication of the antigenic differences between the two clades. Nonetheless, the protection conferred by the NDV-HA and NDV-HAF vaccine viruses was sufficiently broad to protect against both HPAIV challenge viruses.

It was previously observed that replacement of the transmembrane and cytoplasmic tail regions of the H7 HA protein with those of the NDV F protein enhanced incorporation of HA into NDV particles [22]. In that study, enhanced incorporations of the H7 HA protein into NDV particles improved the immunogenicity of the virus. However, this result could not be interpreted because the authors simultaneously change the F protein cleavage site from a mono-basic to a poly-basic motif, which likely would have the effect of increasing replication, spread, and immunogenicity. Nonetheless, only 90% of the vaccinated chickens were protected against challenge with the HPAIV H7 virus. In our study, we have followed the same strategy and examined the incorporation of H5 HA protein into NDV particles. Our Western blot and immune electron microscopy results confirmed that replacement of the transmembrane and cytoplasmic tail domain of H5 HA protein with those of the NDV F protein enhanced incorporation into NDV particles and improved the immunogenicity of the virus by completely protecting (100%) the chickens against NDV and HPAI H5 challenges.

Because expression of the H5 HA protein of a highly pathogenic HPAIV, and in particular its incorporation into the NDV virion, may alter the virulence of the NDV vector, we examined the pathogenicity of the recombinant viruses by the MDT method in embryonated chicken eggs [32]. Our results did not show any increase in pathogenicity of our recombinant viruses compared to that of the parent strain LaSota, which is currently used as a vaccine. However, comparison of growth kinetics of the recombinant virus showed that the virus expressing the chimeric H5 HA protein grew about a log_10_ lower, than the NDV parent or the virus expressing the unmodified H5 HA protein. Similar slow growth kinetics were observed for the virus expressing the chimeric H7 HA protein [22]. It is possible that enhanced incorporation of chimeric HA may have affected the assembly process of NDV.

A concern we wanted to examine is whether enhanced incorporation of the chimeric H5 HA protein may decrease the incorporation of the NDV F protein into the NDV virion. The F protein of NDV is a major immunogenic protein and any reduction in the amount of this protein may alter the efficacy of the NDV vaccine. Analysis of the proteins of purified parental and recombinant viruses by coomassie blue staining did not show any difference in the amount of F protein between parental and recombinant viruses, suggesting that the incorporation of the chimeric H5 HA protein did not take the place of the F protein on the NDV virion (data not shown).

In this study, both of the recombinant NDV vaccine viruses, rNDV-HA and rNDV-HAF, induced similar levels of HI and VN antibodies and provided complete protection against challenges with highly virulent NDV and HPAI H5 viruses. However, further studies involving larger numbers of chickens and additional heterologous challenge viruses will be necessary to determine which of the two vaccine viruses is superior: namely, the NDV-HA virus that replicates somewhat better but incorporates a lesser amount of HPAIV HA, or the NDV-HAF virus that replicates less well (and is more attenuated) but incorporates a greater amount of HA. Our results demonstrated that the NDV-vectored AIV vaccines generated in this study could be used as bivalent vaccines against NDV and HPAI H5 virus in chickens.

## Materials and Methods

### Viruses and cells

Madin-Darby Canine Kidney (MDCK), human epidermoid carcinoma (HEp-2), and chicken embryo fibroblast (DF1) cell lines were obtained from the American Type Culture Collection (ATCC, Manassas, VA). MDCK and HEp-2 cells were grown in Eagle's minimal essential medium (EMEM) containing 10% fetal bovine serum (FBS) and maintained in EMEM with 5% FBS. DF1 cells were grown in Dulbecco's minimal essential medium (DMEM) with 10% FBS and maintained in DMEM with 5% FBS. Recombinant NDV (rNDV) strain LaSota was generated as previously described [31], [34]. The HPAIV strains, A/Vietnam/1203/2004 (H5N1) and A/Hong Kong/213/2006 (H5N1), were obtained from the Centers for Disease Control and Prevention (CDC), Atlanta GA. The HPAIV strain A/Egret/Egypt/01/2006 (H5N1) was obtained from Dr. Daniel Perez and the HPAIV strain H5N2 strain (A/Pennsylvania/8/3/1983) was a field isolate maintained in our virus repository. The viruses were propagated in 9-day-old specific pathogen free (SPF) embryonated chicken eggs.

### Virus titration

The titration of rNDVs and AIVs was carried out by infecting DF1 and MDCK cells, respectively. The rNDV titers were determined by plaque assay in DF1 cells using a 0.8% methylcellulose overlay and 5% allantoic fluid. The infected cells were incubated at 37°C for 3 to 4 days, until the development of plaques was apparent. The cell monolayers were then fixed with methanol and stained with crystal violet for the enumeration of plaques. The HPAIV titers in 50% end point tissue culture infectious dose (TCID_50_) units/ml were determined on monolayers of MDCK cells using the Reed and Muench method [36]. For both NDVs and AIVs, HA titers were determined using chicken RBCs [32]. Egg infective dose 50 (EID_50_) was determined by infecting 5 eggs per group for each 10-fold serial dilution. Following 24 h of infection, eggs were harvested for allantoic fluid and the presence of virus was confirmed by HA test. Similarly, chicken embryo lethal dose 50 (ELD_50_) was determined by observing 50% mortality in embryos at end point dilutions after 48 h of infection. The 50% end point titer was determined by the Reed and Muench method [36]. For HPAIV challenge viruses, the chicken lethal dose of 50 (CLD_50_) was determined by infecting 3 chickens (5-wk-old) per group, and the 50% end point for the lethal dose was determined by the Reed and Muench method [36].

### Generation of rNDVs containing HPAIV HA coding sequence

The construction of a full-length cDNA of the antigenomic RNA of NDV strain LaSota was reported earlier [31], [34]. The present study used a previously-described derivative that had been modified to contain a unique Pme I restriction enzyme site between the P and M genes [31]. The HA open reading frame (ORF) from HPAIV strain A/Vietnam/1203/2004 (H5N1) virus was PCR-amplified using primers (H5HAPmeI sense- 5′-CC*GTTTAAAC* TTA GAAAAAATACGGGTAGAAGGCATA CGCCACCAT GGAG-3′ and antisense 5′-GG*GTTTAAAC*TTAGATACAAATCCT GCACTGG-3′) that introduced an NDV gene junction, including gene end (underlined), intergenic, and gene start (underlined) signals, on the upstream side and Pme I sites (italic) on both sides. The H5 HA gene was further modified to create a second version in which the HA ectodomain was fused to the transmembrane and cytoplasmic domains of the NDV F protein by overlapping PCR using primer H5HAPmeI sense and H5HAF antisense primer (5′-GG*GTTTAAAC*TTAGATACAAATCCTGCACTGG-3′). The cDNA templates used for overlapping PCR were amplified by H5HA PmeI sense primer and antisense primer (5′- GTAATGAGAGCAGATGTGCTTTGGTATATCCCTATGCTCTCC-3′, region corresponding HA is underlined) from H5 HA template and also amplified by sense primer (5′-GGAGAGCATAGGGATATACCAAAGCACATCTGCTCTCATTAC-3′, region corresponding NDV is underlined) and H5HAF antisense from NDV F cDNA template. Altering the transmembrane and cytoplasmic domains changed the length of the HA ORF from 1707 nt to 1767 nt. The PCR products were cloned into full- length NDV plasmid at the unique Pme I site between the P and M genes. The total genome length was maintained as a multiple of six, which is required for efficient NDV replication [35]. In order to decrease the possibility of any mutation or deletion of parts of the NDV genome during propagation in bacteria, the transformed bacteria were grown at 30°C. The HA genes of the resulting plasmids, pNDV-HA and pNDV-HAF, were sequenced to confirm the correct HA gene structure and the absence of any deletion or undesired mutation. Our results showed that the plasmids pNDV-HA and pNDV-HAF were quite stable in bacteria and no mutation or deletion was detected. The NDV recombinants were recovered by transfecting these plasmids along with support plasmids into HEp-2 cells by the previously described procedure [31], [34].

### Expression of H5 HA in cells infected with rNDVs

The HA protein expression by the rNDVs was examined by Western blot and immunofluorescence assays. DF1 cells were infected at a multiplicity of infection (MOI) of 0.01 and were harvested at 48 h post- infection, lysed and analyzed by Western blot using polyclonal chicken H5N1 (post-infection) antiserum. To examine the incorporation of H5 HA and H5 HAF into NDV particles, Western blot analysis was carried out using purified virus and polyclonal chicken H5N1 (post-infection) antiserum. The band intensities in the western blot were normalized using an NDV-HN monoclonal antibody. Immunofluorescence assays were performed to evaluate cell surface and intracellular expression of HA protein. Briefly, DF1 cells were infected with the recombinant viruses at an MOI of 0.01 PFU. At 48 h post-infection, the infected DF1 cells were fixed with 4% para-formaldehyde. For internal H5 HA expression, cells were permeabilized with 0.2% Triton X 100 in PBS for 10 minutes. The primary antibodies used were chicken polyclonal anti-H5 HA and the secondary antibodies used were goat anti-chicken conjugated with FITC. The cells were analyzed with a fluorescent microscope.

### Immunoelectron microscopy

The rNDVs purified by sucrose density gradient from allantoic fluid of infected embryonated eggs were adsorbed to parlodion-filmed carbon-coated nickel grids. The grids were blocked in PBS containing 2% globulin-free BSA and incubated with chicken anti-NDV HN monoclonal antiserum or chicken anti-H5 HA polyclonal antiserum. Grids were washed in blocking solution and incubated in goat anti-chicken or anti- mouse IgG antiserum conjugated to 6 nm gold beads (Aurion, Costerweg 5, The Netherlands). The grids were then negatively stained with 2% ammonium molybdate for 3 min and examined under a transmission electron microscope.

### Growth characteristics of the recombinant viruses in DF1 cells

The growth kinetics of rNDV, rNDV-HA and rNDV-HAF were determined by multicycle growth curves in DF1 cells. DF1 cells in duplicate wells of six-well plates were infected with viruses at a MOI of 0.01 PFU. After 1 h of adsorption, the cells were washed with DMEM and then covered with DMEM containing 5% FBS and 5% allantoic fluid. The cell culture supernatant samples were collected and replaced with an equal volume of fresh medium at 8-hour intervals until 64 hours post-infection. The titers of virus in the samples were quantified by plaque assay in DF1 cells.

### Pathogenicity of recombinant viruses in embryonated chicken eggs

The pathogenicity of the recombinant viruses were determined by the MDT test in 9- day-old embryonated chicken eggs according to a standard protocol [32]. Briefly, 10-fold serial dilutions of fresh infective allantoic fluid ranging from 10^−6^ to 10^−9^ dilutions were made with sterile PBS. 0.1 ml of each diluent was injected into the allantoic cavities of five 9-day-old SPF embryonated chicken eggs (Bee Eggs Company, PA) and incubated at 37°C. Each egg was examined three times daily for 7 days, and times of embryo death were recorded. The minimum lethal dose is the highest virus dilution that causes all embryos inoculated with that dilution to die.

### Immunization and challenge experiments

In experiment one (Table 1), groups of 2-week-old SPF chickens were immunized through the oculonasal route with 10^6^ EID_50_ of biologically-derived wild type NDV LaSota or recombinant virus rNDV (empty vector), rNDV-HA or rNDV-HAF, and an additional group of birds was left uninfected. Three days post-immunization, oropharyngeal (oral) and cloacal swabs were collected for detection of NDV shedding. Three weeks post-immunization, pre-challenge serum samples were collected from all of the birds, and all of the birds were challenged with 10^4^ ELD_50_ of virulent NDV strain Texas-GB through the oculonasal route. Oral and cloacal swabs were collected on day 3 post-challenge for shedding. In experiment two (Tables 2–5), groups of birds were immunized in the same way except that the biologically-derived LaSota control was replaced by its recombinant version rNDV. Three weeks post-immunization, serum samples were collected and the animals were challenged through the intranasal route with 100 CLD_50_ of the homologous HPAIV A/Vietnam/1203/2004 virus or the heterologous HPAIV A/Egret/Egypt/01/2006 virus. To monitor shedding of the challenge viruses, oral and cloacal swabs were collected on day 3 post-infection. Isolation of NDV vaccine vector or HPAIV challenge virus from swabs was carried out by inoculation in 9-day-old embryonated chicken eggs. The chickens were observed daily for 10 days for disease symptoms and mortality, and the presence of NDV or HPAIV was confirmed by HA assay using chicken erythrocytes. Post-challenge serum was collected from the surviving birds before sacrifice after 10 days of challenge. All challenge studies were performed in a BSL3 (Ag) containment facility certified by the USDA and CDC.

### Serological analysis

The antibody levels of serum samples collected from chickens vaccinated with rNDVs were evaluated by hemagglutination (HA), HA inhibition (HI), virus neutralization (VN) and enzyme-linked immunosorbent assays (ELISA) using standard protocols [36]. Cross-reactivity of immunized chicken sera were conducted by VN and HI tests against four homologous and heterologous strains of influenza viruses. The homologous clade 1 H5N1 strains included (A/Vietnam/1203/2004 and A/Hongkong/213/2006), and heterologous strains included clade 2.2 H5N1 virus (A/Egret/Egypt/01/2006) and the H5N2 virus (A/Pennsylvania/8/3/1983). A commercial AIV NP ELISA kit and NDV ELISA kit (Synbiotic Corporation, San Diego, CA) were used to detect antibodies against the NP antigen of type A influenza viruses and whole NDV antigen of NDVs.
